# On Filling Carious Cavities between the Back Teeth

**Published:** 1860-10

**Authors:** Charles Woodnut, W. A. Harris

**Affiliations:** Dentist.; Baltimore.


### ARTICLE XI.

*On Filling Carious Cavities between the Back Teeth.* By CHARLES WOODNUT, Dentist. Translated from the German, by W. A. Harris, M. D., &c. Baltimore.

IT falls to the lot of the dentist to perform a large number of difficult operations, but among them all, there is no one which more frequently disappoints him, than the filling of such cavities as are found between the bicuspids and molars, particularly in the posterior space between the first and second molars. Moreover, precisely in that portion of his work which concerns the filling where it should be firmest, namely, in the upper part of the cavity nearest the gum and beneath the surface of the alveolus, he is apt to fail, because it is impossible for the operator to have a good view of the part, and because he has so small a space under the overhanging roof to manipulate, which is always the case, when the cavity is prepared according to the method delineated in the text-books, a method which has been received by the profession generally, but which is open to many objections; I refer to the operation by which room is secured by a V formed excision, and half the surface of the tooth is removed in such a manner as to prevent its restoration, so that a large portion of the tooth-bone will be deprived of its enamel and exposed to every external corroding agent with which it comes in contact.

The worst feature in this preparatory work, is, that the operator is unable either to examine thoroughly the cavity, or under the most favorable circumstances, to obtain but a very imperfect view; thereby will the success of the operation be so doubtful, that the inexperienced dentist will be undetermined whether to save the tooth or have recourse to immediate extraction. There is a method practiced by some dentists, but, as I understand, not generally known by the profession, which entirely avoids the evils alluded to. My attention will especially be given to the plan adopted by Dr. McQuillen.

With a sharp and strong chisel, that portion of the alveolus which overhangs or forms the roof of the cavity, is cut away, allowing the different soft tissues which connect the cheek, palate or tongue, (according as the tooth involved is above or below,) to remain. After the destroyed mass is removed, and a suitable form is given to the cavity, an artificial passage may be created of greater depth than it was above, so that the cavity from the surface of the alveolus to the neck of the tooth, may be gradually reached. If there be not sufficient room between the tooth and the one adjacent, to condense the filling and continue the work, the respective soft tissues mentioned above, according to the particular necessity, may be detached as far as is required.

The gold, as is usual with a cavity in the crown, is condensed from the alveolus, whilst the upper surface of the same is leveled as usual in these fillings, and assumes a slightly undulating form, so that it cannot easily be displaced by the action of the jaw. Care must be taken that the filling be well consolidated, whilst progress is being made from the bottom of the cavity to the external opening in the intermediate space; above this last the gold must be slightly spread out, to be subsequently condensed. The advantage of this method consists in this, that a cavity of which with great difficulty a limited portion could be seen, and in which there was a small space to manipulate, is changed into a simple crown cavity, most of whose points can be seen, and where in every case direct pressure can be made, so that a more perfect operation is insured, which promises eventual success. The great space, which in this manner is obtained from the jaw, for the benefit of the tooth, counts strongly among the arguments in favor of the operation.

Furthermore, when the pulp is involved posteriorly, the operator is enabled to reach the root with much greater facility and less sacrifice to the substance of the tooth, than by the adoption of any other method, than I have been able to discover.

In regard to this operation, it has been objected that more time and labor would be required to prepare the cavity by this mode, than in the older way; this should not be justly considered an objection if the dentist's duty is better fulfilled, and if, by sharp and properly constructed instruments, the work is finished with greater ease to the operator and more comfort to the patient, as well as in a shorter period of time, than by the old method of filling.

It may be permitted to me to say here a few words in regard to instruments. Many dentists, and particularly the young ones, are accustomed to use instruments which are much too small, and badly proportioned for use; a large heavy handle should not be attached to a small blade, nor a large blade to an insufficient handle. Our instruments must be strong and heavy, but in every case well proportioned. We sometimes use small ones, for instance, in cavities of the incisors, but much the larger portion should be more strongly constructed than those which are generally found in the pocket cases of dentists.

Of much greater importance is it, however, to have sharp instruments, so that our work may be conducted with the most speed, and the least pain for our patients. The dentist can have no excuse for using dull instruments whenever it is possible to obtain a whetstone.

---

### *Arsenic Eating.*

*Messrs. Editors:*

THE noise our papers are making about the eating of this surest of *poisons*, is, we take it, calculated to spread false notions concerning it. The inference is plain, that some parties having had occasion to use arsenic, found its effects pleasant, and continued its use until fear of its ultimate consequences induced them to suspend, when it was found impossible to be omitted, life itself depending upon its continuance. The whole matter must be a fabrication, for there is no approximation to a pleasurable sensation being derived from its use, as in opium or hatchis. That the idea of increasing one's embonpoint would induce any one to resort to this drug, is not true, because its effect after the system has become completely saturated, is simply to give a bloated appearance to the face only, and a bloodshot or inflamed appearance to the eye.

This is absolutely all it can effect, except the invariable consequences when continued, of griping, purging and nausea, and this following the largest dose safely administered of $\frac{1}{10}$ gr. per day.

Fowler's solution of arsenic, the form commonly used of this drug, contains 1.120 of a grain in every drop, and its dose seldom reaches five drops three times daily, or after eating. The whole matter has seemed to me so unreasonable and so widely diffused in the minds of the people, that I have felt justified in uttering a strong protest against it, as great mischief and sad consequences might result.

